# Cost-effectiveness of a clinical decision support system for atrial fibrillation: an RCT-based modelling study

**DOI:** 10.1093/ehjdh/ztaf087

**Published:** 2025-08-01

**Authors:** Olof Persson Lindell, Martin Henriksson, Lars O Karlsson, Staffan Nilsson, Emmanouil Charitakis, Magnus Janzon

**Affiliations:** Department of Cardiology, Linköping University Hospital, Kardiologiska Kliniken, Universitetssjukhuset, Linköping 581 85, Sweden; Department of Health, Medicine and Caring Sciences, Linköping University, Linköping, Sweden; Department of Health, Medicine and Caring Sciences, Center for Medical Technology Assessment, Linköping University, Linköping, Sweden; Department of Cardiology, Linköping University Hospital, Kardiologiska Kliniken, Universitetssjukhuset, Linköping 581 85, Sweden; Department of Health, Medicine and Caring Sciences, Linköping University, Linköping, Sweden; Department of Health, Medicine and Caring Sciences, Linköping University, Linköping, Sweden; Division of Primary Health Care of Region Östergötland, Linköping, Sweden; Department of Cardiology, Linköping University Hospital, Kardiologiska Kliniken, Universitetssjukhuset, Linköping 581 85, Sweden; Department of Health, Medicine and Caring Sciences, Linköping University, Linköping, Sweden; Department of Cardiology, Linköping University Hospital, Kardiologiska Kliniken, Universitetssjukhuset, Linköping 581 85, Sweden; Department of Health, Medicine and Caring Sciences, Linköping University, Linköping, Sweden

**Keywords:** Atrial fibrillation, Clinical decision support, Health care economics, Electronic health record, Cost-effectiveness

## Abstract

**Aims:**

Atrial fibrillation (AF) is a common arrythmia that increases the risk of thromboembolism. Despite the effectiveness of anticoagulation in AF, underuse remains a substantial problem. Clinical decision support (CDS) systems may increase adherence to guideline recommended anticoagulation in AF. However, evidence regarding the cost-effectiveness of these interventions is lacking. The aim of this study was therefore to evaluate the cost-effectiveness of a CDS for AF.

**Methods and results:**

We developed a disease progression model with a Markov structure and simulated a cohort of hypothetical individuals with AF through a standard of care and a CDS strategy. The adherence to anticoagulation in the model was based on the treatment effect reported in the CDS-AF trial, which evaluated the effect of a CDS in patients with AF in the primary care in Östergötland, Sweden. The cost-effectiveness of the CDS-AF intervention compared with standard of care was determined by estimating costs and quality-adjusted life years (QALYs) gained over a lifetime time horizon and was reported as an incremental cost-effectiveness ratio (ICER) assessed against a decision-threshold of €50 000. Uncertainty was evaluated using both one-way and probabilistic sensitivity analysis (PSA). The CDS-intervention resulted in fewer ischaemic strokes but more bleedings. The mean per patient gain in QALYs was 0.012 and the ICER was €963 per QALY gained. The result of the PSA indicated a high probability that the ICER was below €50 000.

**Conclusion:**

The CDS intervention used in the CDS-AF trial appears to yield health gains at a lower cost than typically considered cost-effective.

**Trial registration:**

NCT02635685.

## Graphical Abstract

**Graphical Abstract:**
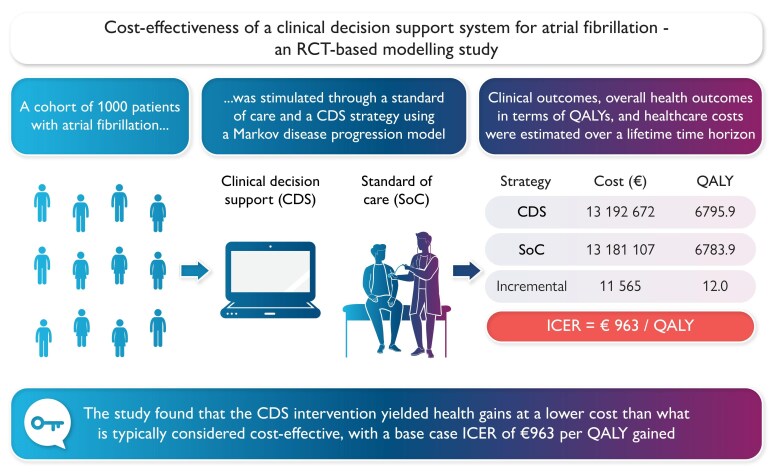
A model-based health economic evaluation of a CDS system, used to increase adherence to guideline recommended anticoagulation therapy in atrial fibrillation. CDS, clinical decision support; ICER, incremental cost-effectiveness ratio; QALY, quality-adjusted life year; SoC, standard of care.

## Introduction

Atrial Fibrillation (AF) is the most common sustained arrythmia, affecting almost 60 million persons globally.^1^ The prevalence of AF is increasing worldwide, and it has been estimated that there will be 14–17 million patients in Europe in 2030.^2,3^ AF increases the risk of thromboembolic events, and AF accounts for ≈ 20% of all strokes.^2,4^ Strokes are associated with a high economic burden and in 32 European countries stroke costs totalled €60 billion in 2017.^5^

Strokes related to AF are to a large extent preventable. Anticoagulation therapy with warfarin can reduce the risk of stroke by more than 60%, and non-vitamin K oral anticoagulants (NOAC) provide an additional risk reduction when compared with warfarin.^6–8^ In the 2020 European Society of Cardiology (ESC) guidelines for the diagnosis and management of AF it was recommended that the CHA_2_DS_2_-VASc clinical stroke risk score be used to identify patients with AF eligible for anticoagulation therapy.^9^

Despite the benefits, underuse of anticoagulation therapy in AF remains a substantial problem, and even if adherence to guidelines has improved, up to 30% of patients are not prescribed oral anticoagulation in line with treatment guidelines.^10–12^

Clinical decision support (CDS) systems are tools that provide patient-specific recommendations to clinicians based on individual patient data in order to aid in decision making.^13^ Previous systematic reviews and meta-analyses have indicated that CDS-systems can improve healthcare performance for a number of diagnoses, but the effectiveness of CDS tools varies and some studies have not been able to show a clear benefit.^14–18^ In addition, there are also potential risks and harms associated with CDS interventions. These risks include disruption of workflow, alert fatigue, and so-called automation bias, where physicians become over dependent on the CDS.^16,19^ Evidence regarding health economic aspects of CDS systems in general is limited, although some studies have concluded that CDS interventions are cost-effective.^13,18,20^

CDS systems have been used in prior studies to improve adherence to anticoagulation guidelines in patients with AF.^21^ The results from these studies are conflicting, where some of the studies have been able to show an increased adherence to guideline recommended anticoagulation treatment while others have not been able to demonstrate any effect of the intervention. Our research group has previously published results from the CDS-AF trial.^22^ In that study we found a significant increase in adherence to guideline anticoagulation after 12 months in the group assigned to a CDS intervention.

Economic outcomes of CDS systems in AF have been sparsely evaluated. The gap in evidence regarding the cost-effectiveness of interventions aimed at increasing anticoagulation use in AF was highlighted in a recent review, where the authors suggested that further research in this area should be a priority as the economic evidence regarding decision support tools in this setting is limited.^23^ This study specifically contributes by providing such evidence. The overall aim of the study was to evaluate the long-term costs and health outcomes of the CDS used in the CDS-AF trial by using well-established decision-analytic modelling methods.

## Methods

### Overview of the economic evaluation

The economic evaluation was based on the CDS-AF trial, a cluster randomized controlled trial (RCT) that evaluated the effects of a CDS system for AF in a primary care setting. A long-term disease progression model was used to evaluate long-term costs and health outcomes of the CDS compared with standard practice (with no CDS). A Swedish healthcare perspective was applied. All costs were expressed in Euros (€) at 2023 prices, and, when necessary, converted from Swedish kronor to € using the average exchange rate in 2023 (1€ = 11.4765 Swedish krona) provided by Sweden’s central bank.^24^ Health outcomes were estimated in quality-adjusted life years (QALYs). Costs and QALYs were discounted by 3% per year according to Swedish guidelines.^25^ An incremental cost-effectiveness ratio (ICER), interpreted as the additional cost per gained QALY if providing CDS rather than standard practice, was calculated. The ICER was compared with the commonly used decision threshold in Sweden of 500 000 SEK (∼€50 000) per QALY.^26^ The model was based on previously published literature on cohort state-transition models in R and developed in R version 4.4.0 (R Core Team, 2024).^27,28^ The development of certain code segments was facilitated by large language model ChatGPT (version 4o).^29^

### Patient population and evaluated strategies

The investigated strategies, CDS and standard of care (SoC), were defined according to the CDS-AF trial (ClinicalTrials.gov NCT02635685).^22^ The CDS-AF trial was conducted in a primary care setting in Region Östergötland (444 347 inhabitants in 2016), Sweden, between January 2016 and January 2017. All primary care clinics in the region (*n* = 43) were randomized 1:1 to CDS intervention or to SoC. The CDS intervention consisted of a CDS system, integrated within the electronic health records (EHR) that was activated in patient with a diagnosis of AF or atrial flutter and a CHA_2_DS_2_VASc score ≥1, without current anticoagulant therapy. The CDS presented an overview of the patient’s diagnosis according to the CHA_2_DS_2_VASc algorithm and estimated the stroke risk for the coming year. The physician could thereafter decide to either prescribe anticoagulation therapy in accordance with guidelines, postpone the decision, or decide to refrain from medication. In the SoC practice the patients were handled according to routine clinical care.

At baseline 14 134 patients with AF were included in the trial, with a mean CHA_2_DS_2_-VASc score of 4. The CDS-AF trial found a significant increase in adherence to guideline recommended anticoagulation therapy in the CDS group vs. the control group after 12 months. In the CDS-group the adherence increased from 70.3% at baseline to 73.0% at the end of the study vs. an increase from 70.0% to 71.2% in the control group (*P* = 0.013), The treatment effect was estimated to be 0.016 [95% CI: 0.003–0.028]. The study did not find any significant differences in the incidence of stroke, transient ischaemic attack, or systemic embolism during the study period of 12 months.^22^

### The disease progression model

**Figure 1:**
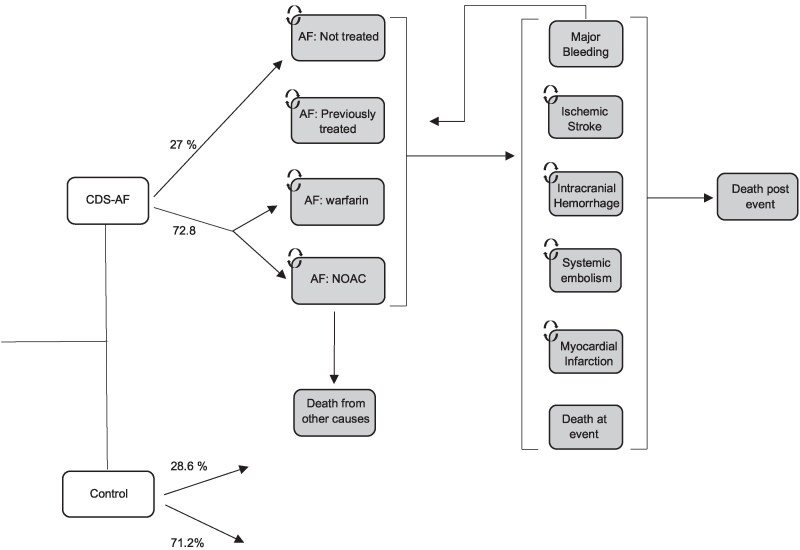
Simplified structure of disease progression model. The first part of the model represents the CDS-AF trial which determined the adherence to guideline recommended anticoagulation therapy in the CDS-group and in the SoC-group. The second part of the model represents the Markov model. In the model the patients start in one of the three health states ‘AF: not treated’, ‘AF: warfarin’ or ‘AF: NOAC’.

Based on the adherence rates for guideline recommended anticoagulation therapy observed in the CDS-AF trial, long-term risk of clinical events was estimated using a disease progression model with a Markov structure (*Figure 1*). In the model, the patients start in an initial health state ‘AF’ where different proportions of patients are treated with anticoagulation therapy in the CDS and the SoC arms, respectively. Each year in the model the patients face a risk of clinical events in the form of ischaemic stroke (IS), intracranial haemorrhage (ICH), extracranial systemic embolism (SE), myocardial infarction (MI) and major bleeding (MB). The risk of these clinical events is conditional on patients being treated with anticoagulation therapy or not.

In all health states in the model patients are accumulating costs and QALYs during each cycle. Some health states are ‘transient’, meaning that the patient only spend one cycle in that health state before moving on to another health state. The first year after all clinical events in the model are transient health states after which the patient moves on to a post-event state in the case of IS, ICH, SE, and MI. After a MB event the patients face a probability of discontinuing or resuming any anticoagulation treatment. After a patient has suffered a clinical event, the patient cannot suffer additional events (except in the case of MB). Hence, only the first event is considered in the case of IS, ICH, SE, and MI, but a worse prognosis is accounted for in the post-event states. During each cycle of the model, the patients face a mortality risk based on age-dependent mortality risk in the general population and state occupancy. A hypothetical cohort of 1000 patients with AF was run through the model for the CDS and SoC strategies, respectively. Half-cycle correction was applied in the analysis of the model that employed a lifetime time horizon to capture the full costs and benefits associated with the CDS-intervention.

#### Data

Input data for the disease progression model were based on the CDS-AF trial and previously published literature. A summary of key input parameters of the model is presented below. Details of all data inputs are available in the Supplementary material.

#### Disease progression

Long-term annual risk rates of clinical events were sourced from previously published studies and used to estimate annual transition probabilities in the model. We applied a baseline adherence to guideline recommended anticoagulation therapy of 72.8% in the CDS-group and 71.2% in the SoC-group, in accordance with the treatment effect size found in the CDS-AF trial. Data from the National Board of Health and Welfare was used to estimate the proportion of patients on warfarin and NOAC-treatment, respectively.^30^ However, the difference in adherence between the groups that was found in the CDS-AF trial (1.6% points increased adherence in the intervention cohort) was assumed to be constituted of patients treated with NOAC, since NOAC-therapy was the first-line treatment recommended in our region at the time of the study. The annual risk of IS in untreated individuals was estimated using a study by Friberg *et al*.^31^ The annual stroke risk of patients with a CHA_2_DS_2_-VASc score of 4 points was used in the model as this score best matched the CHA_2_DS_2_-VASc score of the patients in the CDS-AF trial. The starting age of the simulated cohort was 75 years. Key disease progression parameter values are summarized in Supplementary material online, *Table S1*.

#### Health-related quality of life

In the disease progression model, QALY weights were assigned to each health state to reflect the health-related quality of life associated with that particular state. Age-specific QALY weights for the general population were sourced from a previously published study of the general population in Sweden.^32^ These QALY weights were used for the patients in the model, since it has been shown that in the absence of comorbidities long-term AF has negligible impact on quality of life in elderly patients.^33,34^ In addition, this assumption was not expected to affect the overall results of the analysis. A small QALY decrement was added to AF-patients treated with anticoagulation therapy,^35^ with similar decrements applied for warfarin and NOAC treated patients.^36,37^ QALY decrements following clinical events were estimated based on data from previously published studies. All QALY weights are presented in Supplementary material online, *Table S2*.

#### Costs

To determine the cost of using the CDS, we used data from the CDS-AF trial. We estimated the average time needed to manage a CDS pop-up by using our best clinical judgment. We also estimated the time required to initiate anticoagulation therapy and inform the patient in cases where it was required. The validity of these estimates was verified with several external primary care physicians, not part of the study group. The unit costs applied to the working hours consumed were estimated using the average Swedish primary care physician salary in 2023 obtained from the Swedish Confederation of Professional Associations, including employer’s contributions.^38^ We also registered the annual fees associated with using the CDS. This information was obtained directly from the information technology department of Region Östergötland. The sum of these costs was divided by the number of AF-patients to calculate a cost per patient. The one-time cost for development and implementation of the CDS was not incorporated into the disease progression model in the base case. We estimated these costs to ∼€185 000. The costs of clinical events and the costs related to anticoagulation therapy were estimated based on data from previously published studies. All costs are presented in Supplementary material online, *Table S3*.

### Sensitivity analysis

We conducted a probabilistic sensitivity analysis (PSA) to account for uncertainty in the input parameters of the disease progression model. In the PSA, the uncertainty in model inputs was characterized as probability distributions that were propagated through the model in 10 000 simulations to provide a probability distribution of the cost-effectiveness estimate. For relative risk parameters, the uncertainty of the parameters in the PSA was estimated based on confidence intervals (CI) provided from the source literature. For transition rates, utility decrements, and costs, distributional information was generally lacking, and we assumed that the standard error was ¼ of the mean. In addition to the PSA, we also perform one-way sensitivity analyses to estimate the impact of changes in individual parameters included in the model. Different scenarios were analyzed by varying the relevant parameters inputs, these scenarios included changing the discount rate, changing the treatment effect of the CDS, and changing the underlying risks of clinical events. The parameters were reduced by 50% and increased by 200% in most cases. When dealing with proportions, the parameters were adjusted to ensure that the parameters remained between 0 and 1. Additional analyses were performed for the most important model parameters by investigating how a wider range of input values were related to the results of the model. Finally, we undertook exploratory analyses to investigate cost-effectiveness of the CDS-AF system in populations with other risk profiles.

### Presentation of results

The results of the cost-effectiveness analysis were presented as an ICER, where the incremental costs of the CDS compared with SoC are divided by the incremental QALYs. From the PSA, we illustrate the distribution of the cost-effectiveness results by plotting the incremental costs and QALY pairs from the simulations on the cost-effectiveness plane. The one-way sensitivity analyses were presented in a tornado diagram. Furthermore, we present the number of clinical events estimated by our disease progression model. These results enable the clinical reader to understand the overall behaviour of the model and validate the clinical plausibility of the model.

### Ethical considerations

The main ethical considerations related to the CDS system used in the CDS-AF trial have been published previously.^22,39^ The CDS-AF trial received ethical approval by the Regional Ethical Review Board of Linköping (Dnr: 2015/204-31). In that application it was specified that a health economic evaluation was to be performed based on the trial data.

### Consent

The Regional Ethical Review Board of Linköping waived the requirement of informed consent and approved the study.^22^

## Results

### Base case

The results of the base case analysis are presented in *Table 1*. For the cohort of 1000 patients, over a lifetime time horizon, the CDS-intervention resulted in fewer ISs but more MBs. There were minor changes in the number of MIs, SEs and ICHs. The CDS yielded an additional cost of anticoagulation treatment, on top of the cost of the CDS itself. However, in the CDS-group there was a reduction in event-related costs. In total, the discounted costs were slightly higher in the CDS group when compared with SoC. Total discounted QALYs were higher in the CDS group and for the cohort, 12 QALYs were gained. In total the simulation resulted in an ICER of €963 per QALY gained from the CDS-intervention.

**Table 1:** Base case analysis

Number of events	Standard of care	Clinical decision support	Incremental
Ischaemic Strokes	242.34	239.10	−3.25
Intracranial haemorrhages	16.66	16.84	+0.18
Myocardial infarctions	125.04	124.33	−0.71
Systemic embolisms	34.62	33.93	−0.69
Major bleedings	143.74	145.21	+1.47
Cost-effectiveness			
Cost of CDS	€0	€2996	€2996
Cost of anticoagulation treatment	€4 629 728	€4 739 269	€109 541
Cost of clinical events^a^	€8 551 379	€8 450 407	€−100 972
Total costs	€13 181 107	€13 192 672	€11 565 (€11.6 per patient)
Total QALYs	6783.9	6795.9	12.0 (0.012 per patient)
Cost/QALY gained			€963

Predicted number of undiscounted clinical events per 1000 AF-patients and discounted cost-effectiveness results for CDS-intervention vs. SoC.

^a^Accumulated costs of all years spent in post-event health states.

### Probabilistic sensitivity analysis

**Figure 2:**
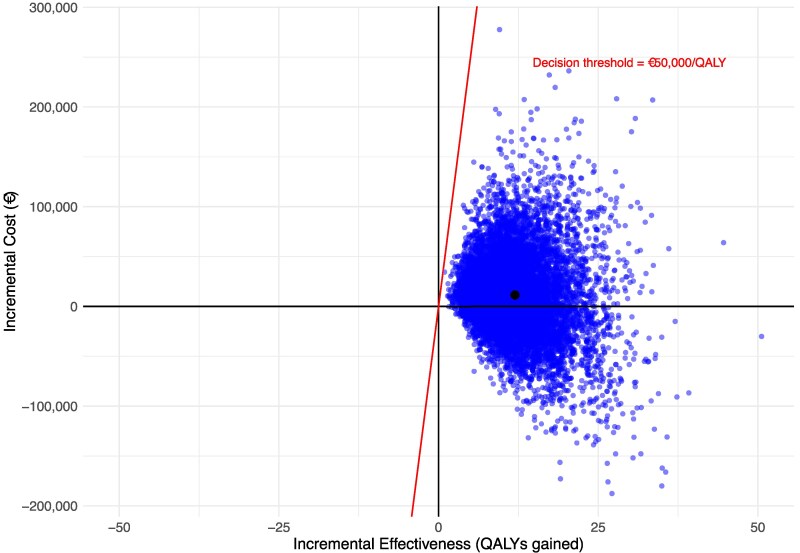
Cost-effectiveness plane. The incremental cost-effectiveness plane of 10 000 Monte Carlo simulations, showing incremental costs and incremental effectiveness of the CDS as compared to SoC. All points below the red decision threshold line have an estimated ICER < €50 000 per QALY gained. The black point is the mean result of all simulations.

The result of the PSA analysis is presented in *Figure 2*. The mean ICER estimated from 10 000 Monte Carlo simulations was €956 per QALY gained. The CDS intervention was associated with a cost per QALY below €50 000 in 100% of the simulations. The CDS intervention was dominant (lower costs and improved QALYs) in 38.5% of the simulations.

### One-way sensitivity analysis

**Figure 3:**
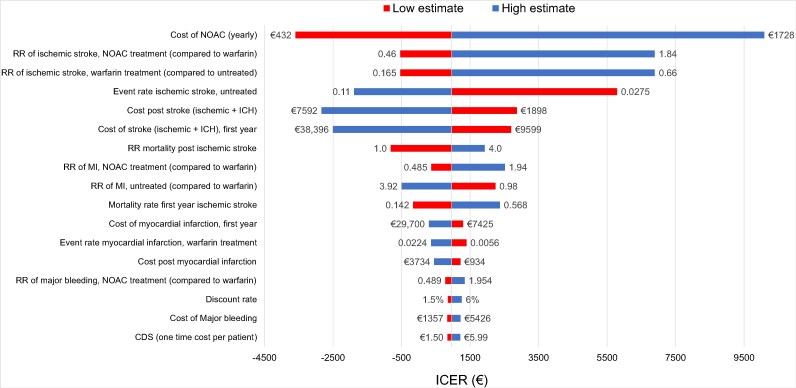
One-way sensitivity analysis. The figure shows the effect of isolated changes in single parameters on ICER. The ICER in the base case was €963 per QALY gained. The value next to each bar represents the highest and lowest values that were used for each parameter in the analysis. Note that a negative ICER in this graph indicates that CDS dominates SoC.

**Figure 4:**
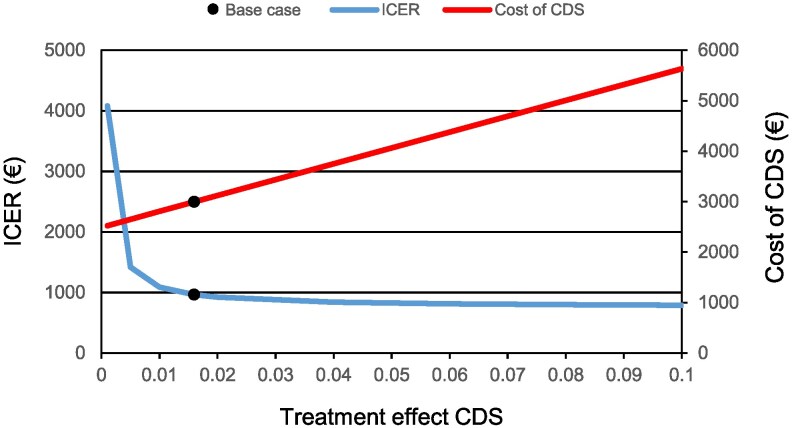
Sensitivity analysis of CDS-treatment effect. The figure shows the uncertainty of isolated changes in the CDS-treatment effect on ICER, and costs related to the CDS. The black point is the base case scenario.

The results of the one-way sensitivity analysis are presented in *Figure 3*. The cost-effectiveness of the CDS-intervention was most sensitive to changes in the yearly cost of NOAC-treatment, followed by the relative risk reduction of anticoagulation treatment on the risk of IS, and the underlying yearly event rate of IS in untreated individuals. The cost per QALY gained with the CDS-intervention remained below a decision threshold of €50 000 per QALY. When changing the time horizon of the analysis to 5, 10, 15 and 20 years, the ICER was below the decision threshold in all cases (€9119, €1765, €925, and €921, respectively). The uncertainty related to the CDS-treatment effect was further evaluated by varying the treatment effect of the CDS between 0.001 and 0.1. This analysis showed that the cost of the CDS increased linearly while the ICER fell progressively with a higher treatment effect (*Figure 4*). As a separate analysis we also analyzed the cost-effectiveness of the CDS intervention when including the costs for developing and implementing the CDS (€185 000). In this scenario, the cost of using the CDS increased to €16.2 per patient and to €16 184 for the entire cohort, as compared to €3.0 and €2996, respectively, in the base case scenario. The ICER of the CDS-intervention increased to €2062 compared with €963 in the base case. We further analyzed the highest acceptable cost for the CDS in order for the intervention to maintain an ICER below the decision threshold and found the upper limit to be €592 per patient or €591 570 in total for the cohort.

Finally, analyses were performed to investigate the cost-effectiveness of the CDS-AF in populations with other risk profiles. In the analyses we adjusted the annual event rates of IS and SE to match a cohort with a lower embolic risk, similar to patients with a CHA_2_DS_2_-VASc score of 1 (Scenario 1) and a higher CHA_2_DS_2_-VASc score of 6 (Scenario 2). In Scenario 1, we ran the model for 65-year olds instead of 75 years, and did not allow the event rates of IS and SE to change during the simulation (i.e. -the rates were not altered when patients turned 75 years in the model and thereby would have reached a CHA_2_DS_2_-VASc score of at least 2). In Scenario 1, we also reduced the event rates of MI, ICH and MB to one-third of the base case rates to more accurately reflect a healthier population. Scenario 1 resulted in an ICER of €46 454 per QALY gained. In Scenario 2, when increasing rather than reducing the event rates of IS and SE -the CDS intervention was a dominant strategy.

## Discussion

In this economic evaluation of the CDS-AF trial, we simulated a hypothetical cohort of 1000 AF-patients in a disease progression model, using a life-time time horizon. The main finding was that the CDS-intervention was associated with a cost of €963 per QALY gained compared with SoC. The model predicted that the CDS-intervention would result in fewer ISs but cause more MBs. The results further show a high probability that the cost per QALY gained with CDS is well below generally accepted decision thresholds in Sweden; 100% of the simulations when applying a threshold of €50 000. Our model predicted a modest gain in QALYs per patient (0.012 QALY), but this was achieved at a low incremental cost associated with the CDS intervention. To the best of our knowledge, this is the first study to present a thorough health economic evaluation of a CDS tool used to increase adherence to guideline recommended anticoagulation treatment in AF. As such, this is the first study to provide cost-effectiveness evidence to decision-makers who are responsible for deciding whether to use these tools or not.

As previously mentioned, several studies have evaluated the use of CDS systems in patients with AF, but economic evaluations of these interventions are limited. In one study the authors presented some cost-related information regarding their CDS intervention.^40^ In that study, the authors implemented a computer-based alert system to increase anticoagulation prescription and found that the intervention modestly improved the prescription rate. No full economic evaluation was reported, but the authors stated that the cost of the entire project was $230 000 (€270,000, converted to euros at 2023 prices), and that most of the costs were related to the development and testing of the CDS. That number is slightly above the cost of developing and implementing the CDS system in our study (∼€185 000).

A recent review highlighted the lack of economic evidence regarding interventions designed to increase anticoagulation use in AF, and called for further research in this area.^23^ Previous health economic evaluations have mainly focused on other types of interventions to improve adherence to guideline recommendations in AF. Hendriks *et al.*^41^ compared an intervention consisting of a nurse-led clinic supported by a decision tool to usual care for the management of AF in terms of anticoagulation treatment, rate/rhythm control and cardiovascular risk management.^41^ They found that the intervention resulted in a higher mean total QALY and a lower total health-care cost per patient in the nurse-led group in a trial-based evaluation with a 12-month duration. The gain in QALYs was not statistically significant, but the authors concluded that the nurse-led clinic was a dominant strategy.

In a previous study by Blomström Lundqvist *et al.*^42^ the authors investigated the cost-effectiveness of two interventions to improve patient adherence in AF.^42^ Lower patient adherence was found to be associated with an increased number of IS events, increased mortality, and reduced quality of life. The study also found that the two interventions (patient education and chronic disease co-management) could be cost-effective or even cost saving.

Several previous health economic studies have investigated the cost-effectiveness of screening for AF, demonstrating results ranging from dominant to ICERs of €39 485 per QALY gained, when screening individuals 75 years or older.^43^ When comparing interventions that screen for AF with this CDS intervention that focused on increasing adherence to guideline recommended anticoagulation therapy in patients already diagnosed with AF, the ICERs seem to indicate a somewhat similar cost-effectiveness, achieved at low per patient QALY gains and low incremental costs.

CDS systems have also been used in other areas of cardiovascular medicine and in a recent review the authors found mixed results regarding improvement in cardiovascular disease related clinical outcomes, and concluded that there was a lack of evidence regarding economic outcomes.^18^ Of the 58 RCTs included in the review, only six reported any economic outcomes and of these only two reported on the cost-effectiveness of the interventions. Hence, there is not only a lack of health economic evaluations of CDS systems in AF but a lack of economic evaluations of CDS-systems in cardiovascular medicine in general. Our study provides an attempt to close this large gap in knowledge.

Finally, the future role of CDS systems should be seen in the context of other emerging technologies. In the past, CDS systems have mainly relied on knowledge based, rule-driven algorithms.^17,44^ However, in later years, the potential role of artificial intelligence (AI) to power CDS systems has been highlighted.^16,45^ AI-based tools have already been developed to aid in AF detection and anticoagulation treatment decisions.^46^ Further, some AI-based algorithms have been shown to be superior to classical clinical risk scores such as the CHA_2_DS_2_-VASc score and HAS-BLED score in predicting stroke and bleeding risk in patients with AF^46–48^ AI in this context appears promising, but these systems should be thoroughly evaluated like any other system, before broad clinical implementation. Our disease progression model, or other similar models, serve as a framework for evaluating the cost-effectiveness of these AI-based CDS systems.

### Limitations

As mentioned previously, we used data from the CDS-AF trial to determine the adherence to anticoagulation therapy used in the model. It is possible that the effectiveness of the CDS system is related to the proportion of patients that are already prescribed anticoanticoagulation therapy. Hence, it is possible that the effectiveness of the CDS could be even higher in other populations, since the AF-population in the CDS-AF trial already had an adherence to guideline anticoagulation of ≈ 70% at baseline. The impact of the treatment effect on the cost-effectiveness was evaluated in a sensitivity analysis that indicated a modest reduction in the ICER with an increasing treatment effect. Further, in our model we did not take into account that the CDS might continue to improve adherence after the first year. The long-term upper limit of adherence that is achievable by using the CDS-AF is yet to be known. Since we have no clinical data on the effectiveness of the CDS after 1 year, this model assumed that the CDS did not have any effect on adherence after the first year.

The cost-effectiveness of the CDS-AF system appears to be sensitive to the underlying risk profile of the targeted population, as seen in our sensitivity analyses. When dealing with a population at low risk of embolic events the ICER approached the generally accepted decision threshold in Sweden. However, when dealing with a high-risk population the intervention was in contrast dominant. Therefore, before adopting a CDS similar to the CDS-AF, it is important to not only consider the underlying adherence to guideline anticoagulation therapy in the population, but also the underlying risk profile.

In the base case scenario, the costs of developing and implementing the CDS were not included. These could be considered sunk costs if the decision problem is to consider keeping the decision support system. However, since costs of implementing the CDS will be incurred if introduced in another setting, we included them in a separate sensitivity analysis. This analysis indicated that the impact on the overall cost-effectiveness was small. Furthermore, it should be noted that in some cases the upfront costs related to implementing the CDS are difficult to estimate, not least because it is difficult to estimate the impact on resource constraints (i.e. personnel time).

The CDS used in the CDS-AF trial used the CHA_2_DS_2_-VASc risk score to identify patients eligible for anticoagulation therapy. This risk score has recently been revised, and the ESC guidelines now recommends using the updated CHA_2_DS_2_-VA score.^1^ One could argue that the CDS used in the CDS-AF trial is somewhat outdated since the CHA_2_DS_2_-VASc score is no longer recommended in clinical practice. However, the CDS-AF trial used different scoring levels for men and women to qualify for anticoagulation therapy. Therefore, there would be no practical difference in terms of whether anticoagulation therapy would be recommended or not, regardless of which of the two risk scores that was used.

In our model we did not take into account treatment discontinuation. However, one could argue that it is not likely that there will be a large difference between the groups in this regard and therefore we did not expect this to have a large impact on the overall cost-effectiveness of the CDS.

Finally, the health economic analysis in this study was conducted using a Swedish health care perspective. Translating these results to other health care settings should be done with caution, as different healthcare systems may vary on several parameters, including, but not limited to, the underlying adherence to guideline recommended anticoagulation therapy, the risk profile of the population, cost-structures, and the use of different EHRs. However, for healthcare systems with similar set up as the Swedish, most inputs should be valid, and the results generalisable. If this is not the case, the study provides a framework for evaluation where inputs can be amended to fit other healthcare systems.

## Conclusion

This model-based health economic evaluation predicted that the CDS-AF intervention resulted in less strokes but more major bleedings when compared with SoC. The intervention yielded health gains at a lower cost than what is typically considered acceptable in the Swedish healthcare system, with a base case ICER of €963 per QALY gained. The implication for health care policy is that for a cohort of 1000 patients 12 years of full health can be gained at an incremental cost of ∼€12 000.

## Lead author biography


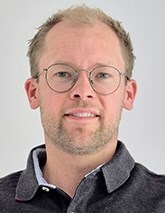


Olof Persson Lindell is a medical doctor at the department of Cardiology at Linköping University Hospital, Linköping, Sweden and a PhD-student at the Department of Health, Medicine and Caring Sciences (HMV), at Linköping University, Sweden. He earned his MD degree from Karolinska Institutet, Stockholm, Sweden, in 2014. He has completed internal medicine training and is currently undergoing cardiology training. Olofs research is focused on clinical decision support systems in cardiovascular disease and health economics. His main supervisors are associate professor Magnus Janzon, associate professor Lars O Karlsson and associate professor Martin Henriksson.

## Supplementary Material

ztaf087_Supplementary_Data

## Data Availability

The data underlying this article will be shared on reasonable request to the corresponding author.
