# Supplementary material for: Cost-effectiveness of a clinical decision support system for atrial fibrillation: an RCT-based modelling study
Source: Eur Heart J Digit Health. 2025 Aug 1;6(5):997–1005. doi: 10.1093/ehjdh/ztaf087 (PMC12450513; doi:10.1093/ehjdh/ztaf087)
Supplement: ztaf087_Supplementary_Data [file ztaf087_supplementary_data.docx]

# Supplemental information to “Cost-effectiveness of a clinical decision support system for atrial fibrillation – an RCT-based modelling study”

| Supplementary table 1. Key disease progression parameter input. | | | | |  |
| --- | --- | --- | --- | --- | --- |
| Parameter | Mean/Base-Case | High-Low in sensitivity analysis | Distribution in PSA | Source | |
| Treatment effect of CDS-AF | 0.016 | 0.008 - 0.032 | Beta (6.98, 429.35) | (1) | |
| Proportion of patients receiving anticoagulation SoC | 0.712 | 0.512 - 0.912 † | Beta (7120, 2880) | (1) | |
| Proportion of patients on warfarin | 0.21* | 0.105 - 0.42 | Beta (2100, 7900) | (2) | |
| Event rate ischemic stroke, untreated | 0.055 | 0.0275 - 0.11 | Gamma (16, 0.00344) | (3) | |
| RR of ischemic stroke, warfarin treatment (compared to untreated) | 0.33 | 0.165 - 0.66 | Lognormal (-1.109, 0.184) | (4) | |
| RR of ischemic stroke, NOAC treatment (compared to warfarin) | 0.92 | 0.46 - 1.84 | Lognormal (-0.0834, 0.0526) | (5) | |
| Mortality rate first year ischemic stroke | 0.284 | 0.142 - 0.568 | Gamma (16, 0.0178) | (6) | |
| RR mortality post ischemic stroke | 2.0 | 1.0 - 4.0 | Lognormal (-0.693, 0.114) | (7) | |
| Event rate ICH, warfarin treatment | 0.0038 | 0.0019 - 0.0076 | Gamma (16, 0.000238) | (8) | |
| RR of ICH, untreated (compared to warfarin) | 0.22 | 0.11 - 0.44 | Lognormal (-1.514, 0.584) | (9) | |
| RR of ICH, NOAC treatment (compared to warfarin) | 0.48 | 0.24 - 0.96 | Lognormal (-0.734, 0.106) | (5) | |
| Mortality rate first year ICH, untreated | 0.693 | 0.303 - 1.21 | Gamma (16, 0.0378) | (10) | |
| RR Mortality first year ICH on anticoagulation | 1.18 | 0.59 - 2.36 | Lognormal 0.166, 0.0294) | (10) | |
| RR mortality post ICH | 2.0 | 1.0 - 4.0 | Lognormal (-0.693, 0.114) | assumption | |
| Event rate myocardial infarction, warfarin treatment | 0.0112 | 0.0056 - 0.0224 | Gamma (16, 0.0007) | (11) | |
| RR of MI, untreated (compared to warfarin) | 1.96 | 0.98 - 3.92 | Lognormal (0.673, 0.420) | (9) | |
| RR of MI, NOAC treatment (compared to warfarin) | 0.97 | 0.485 - 1.94 | Lognormal (-0.0305, 0.111) | (5) | |
| Mortality rate first year, myocardial infarction | 0.206 | 0.103- 0.412 | Gamma (16, 0.01286) | (12) | |
| RR Mortality, post MI | 2,0 | 1.0 - 4.0 | Lognormal (-0.693, 0.114) | (13) | |
| Event rate systemic embolism, untreated | 0.00897 | 0.00449 - 0.0179 | Gamma (16, 0.000560) | (3, 14) | |
| RR of systemic embolism, warfarin treatment (compared to untreated) | 0.29 | 0.145 - 0.58 | Lognormal (-1.238, 0.666) | (15) | |
| RR of systemic embolism, NOAC treatment (compared to warfarin) | 0.68 | 0.34 - 1.36 | Lognormal (-0.386, 0.178) | (16) | |
| Mortality rate first year, systemic embolism | 0.284 | 0.142 - 0.568 | Gamma (16, 0.0178) | (6, 14) | |
| RR mortality post systemic embolism | 1.2754 | 0.638 - 2.551 | Lognormal (0.243, 0.176) | (6, 14) | |
| Event rate major bleeding, warfarin | 0.0182 | 0.0091 - 0.0364 | Gamma (16, 0.00114) | (8) | |
| RR of major bleeding, untreated (compared to warfarin) | 0.60 | 0.3 - 1.2 | Lognormal (-0.511, 0.362) | (4) | |
| RR of major bleeding, NOAC treatment (compared to warfarin) | 0.977 | 0.489 - 1.954 | Lognormal -0.0233, 0.0464) | (17) | |
| Mortality rate major bleeding (untreated, NOAC, warfarin) | 0.03 | 0.015 - 0.06 | Gamma (16, 0.00188) | (18) | |
| Probability of not resuming anticoagulation after major bleeding | 0.53 | 0.23 - 0.83 † | Beta (6.99, 6.199) | (19, 20) | |
| Abbreviations: CDS = clinical decision support, ICH = intracranial hemorrhage, MI = myocardial infarction, NOAC = non-vitamin K oral anticoagulants, PSA = probabilistic sensitivity analysis, RR = relative risk SoC = standard of care. All event rates are annual rates. * The difference in adherence between standard of care and intervention group (1,6 % of population in the intervention group) was assumed to receive NOAC. † High/Low value was adjusted to ensure that the parameter remained between 0-1. | | | | |  |

| Supplementary table 2. QALY-weights applied in model. | | | |  |  |  |
| --- | --- | --- | --- | --- | --- | --- |
| Parameter | Mean/Base-case | High-Low in sensitivity analysis | Distribution in PSA | | Source | |
| QALY weight stable atrial fibrillation | same as general population | Fixed | Fixed | | (21-23) | |
| QALY-decrement anticoagulation treatment | 0.013 | 0.0065 - 0.026 | Gamma (16, 0.000813) | | (24) | |
| QALY-decrement ischemic stroke, first year | 0.160 | 0.08 - 0.32 | Gamma (16, 0.01) | | (25) | |
| QALY-decrement post ischemic stroke | 0.185 | 0.0925 - 0.37 | Gamma (16, 0.0116) | | (25) | |
| QALY-decrement ICH, first year | 0.2125 | 0.106 - 0.425 | Gamma (16, 0.0133) | | (25) | |
| QALY-decrement post ICH | 0.0688 | 0.034 - 0.138 | Gamma (16, 0.0043) | | (25) | |
| QALY-decrement all myocardial infarction states | 0.0627 | 0.0314 - 0.125 | Gamma (16, 0.00392) | | (26) | |
| QALY-decrement all systemic embolism states | 0.0508 | 0.0254 - 0.101 | Gamma (16, 0.00318) | | (27) | |
| QALY-decrement major bleeding | 0.0174 | 0.0087 - 0.0348 | Gamma (16, 0.00109) | | (28) | |
| Abbreviations: ICH = intracranial hemorrhage, PSA = probabilistic sensitivity analysis, QALY = quality adjusted life year | | | | | |  |

| Supplementary table 3. Costs applied in model. | | | | |  |
| --- | --- | --- | --- | --- | --- |
| Parameter | Mean/Base-case | High-Low in sensitivity analysis | Distribution in PSA | Source | |
| Stroke, first year (ischemic + ICH) | €19,198 | €9599- 38,396 | Gamma (16, 1199.875) | (29) | |
| Post stroke (ischemic + ICH) | €3796 | €1898 - 7592 | Gamma (16, 237.25) | (29) | |
| Myocardial infarction, first year | €14,850 | €7425 – 29,700 | Gamma (16, 928.125) | (30) | |
| Post myocardial infarction | €1867 | €934 - 3734 | Gamma (16, 116.6875) | (30) | |
| Systemic embolism, first year | €4664 | €2332 - 9328 | Gamma (16, 291.5) | (31) | |
| Post systemic embolism | €864 | €432 - 1728 | Gamma (16, 54) | assumption | |
| Major bleeding | €2713 | €1356.5 - 5426 | Gamma (16, 169.562) | (31) | |
| Warfarin (yearly cost) | €669 | €334.5 - 1338 | Gamma (16, 41.8125) | (32-35) | |
| NOAC (yearly cost) | €864 | €432 - 1728 | Gamma (16, 54) | (33-35) | |
| CDS (total one-time cost per patient) | €3.0 | €1.50- 5.99 | Individual parameters varied † | estimate * | |
| Abbreviations: CDS = clinical decision support, ICH = intracranial hemorrhage, NOAC = non-vitamin K oral anticoagulants, PSA = probabilistic sensitivity analysis. *Estimated by the research group as described in the article, †The one-time cost per patient for using the CDS was a sum of several different parameters that were all varied in the PSA | | | | |  |

## Clarification of all parameters in the model

### Disease progression model parameter input

### Time-horizon

Base case: Lifetime (model run from starting age of 75 to 105 year).

One-way sensitivity analysis: 5, 10, 15, 20 years

Distribution in PSA: Fixed at lifetime in PSA

Source: Not Applicable (N/A)

### Discount rate

Base case: 0.03

One-way sensitivity analysis: 0.015 - 0.06

Distribution in PSA: Fixed at 0.03 in PSA

Source: (36)

### Treatment effect of CDS-AF

Base case: 0.016

One-way sensitivity analysis: 0.008 - 0.032

Distribution in PSA: Beta (6.98, 429.35)

Source: (1)

### Proportion of patients receiving anticoagulation in Standard of Care (SoC)

Base case: 0.712

One-way sensitivity analysis: 0.512 - 0.912. This parameter was only varied ± 0.2 instead of -50 % +200 % to ensure that the parameter remained between 0-1.

Distribution in PSA: Beta (7120, 2880)

Source: (1)

### Proportion of patients on warfarin

Base case: 0.21. For the base case we used data from the National Board of Health and Welfare to estimate the proportion of patients on warfarin and NOAC treatment, respectively. In total 21 % was found to be treated with warfarin. We assumed in the model that the rest would be treated with NOAC and used the average combined treatment effect of NOACs for all transition probabilities. In the case of the clinical decision support (CDS) group we assumed that the difference in adherence to anticoagulation therapy between the groups that was found in the CDS-AF study (1,6 percentage points increased adherence in the intervention cohort) was constituted of patients treated with NOAC, since NOAC-therapy was the first-line treatment recommended in our region at the time of the study.

One-way sensitivity analysis: 0.105 - 0.42

Distribution in PSA: Beta (2100, 7900)

Source: (2)

### Age-specific mortality rate general population

Base case: The underlying risk of death used in the model was sourced from life tables of the year 2023 provided by Statistics Sweden. We used the average rate of men and women.

One-way sensitivity analysis: Fixed

Distribution in PSA: Fixed

Source: (37)

### Event rate ischemic stroke, untreated

Base case: 0.055. The annual ischemic stroke (IS) risk of patients with a CHA_2_DS_2_-VASc score of 4 points was used in the model as this score best matched the CHA_2_DS_2_-VASc score of the patients in the CDS-AF study.

One-way sensitivity analysis: 0.0275 - 0.11

Distribution in PSA: Gamma (16, 0.00344)

Source: (3)

### RR of ischemic stroke, warfarin treatment (compared to untreated)

Base case: 0.33. Based on a study by Hart et al that found that warfarin was associated with a 67 % (CI, 54% to 77%) relative risk reduction of IS.

One-way sensitivity analysis: 0.165 - 0.66

Distribution in PSA: Lognormal (-1.109, 0.184)

Source: (4)

### RR of ischemic stroke, NOAC treatment (compared to warfarin)

Base case: 0.92. The relative risk of IS in patients treated with NOAC compared to patients treated with warfarin was obtained from a previously published meta-analysis by Ruff et al. We used data for “Pooled NOAC” presented in the article.

One-way sensitivity analysis: 0.46 - 1.84

Distribution in PSA: Lognormal (-0.0834, 0.0526)

Source: (5)

### Mortality rate first year ischemic stroke

Base case: 0.284. The mortality rate during the first year after IS in patients with atrial fibrillation was obtained from a previously published study by Vinding et al. We assumed that the mortality rate after IS was the same independent of whether patients were treated with anticoagulation therapy or not.

One-way sensitivity analysis: 0.142 - 0.568

Distribution in PSA: Gamma (16, 0.0178)

Source: (6)

### RR mortality post ischemic stroke

Base case: 2.0. The relative risk of long-term mortality after IS was obtained from a previous study by Fang et al.

One-way sensitivity analysis: 1.0 - 4.0

Distribution in PSA: Lognormal (-0.693, 0.114)

Source: (7)

### Event rate ICH, warfarin treatment

Base case: 0.0038. The annual risk of intracranial hemorrhage (ICH) in patients treated with warfarin was obtained from a study by Sjögren et al.

One-way sensitivity analysis: 0.0019 - 0.0076

Distribution in PSA: Gamma (16, 0.000238)

Source: (8)

### RR of ICH, untreated (compared to warfarin)

Base case: 0.22. The relative risk of ICH in patients not treated with any anticoagulation compared to warfarin treated was obtained from a meta-analysis presented by Tawfik et al. The value presented in the base-case mixed-treatment comparison analysis was used.

One-way sensitivity analysis: 0.11 - 0.44

Distribution in PSA: Lognormal (-1.514, 0.584)

Source: (9)

### RR of ICH, NOAC treatment (compared to warfarin)

Base case: 0.48. The relative risk of ICH in patients treated with NOAC compared to warfarin treated was obtained from a meta-analysis presented by Ruff et al.

One-way sensitivity analysis: 0.24 - 0.96

Distribution in PSA: Lognormal (-0.734, 0.106)

Source: (5)

### Mortality rate first year ICH, untreated

Base case: 0.693. The mortality rate the first year after ICH was obtained from a study by Fernando et al. This cohort included both patients with and without atrial fibrillation (10,8 % had AF or atrial flutter), but we assumed that the mortality rate after ICH would be similar in patients with and without AF. The authors present the mortality (%) at 1 year in patients without OAC use (50.0%). The mortality (%) was converted to a 1-year mortality rate.

One-way sensitivity analysis: 0.303 - 1.21

Distribution in PSA: Gamma (16, 0.0378)

Source: (10)

### RR Mortality first year ICH on anticoagulation

Base case: 1.18. The relative risk of mortality in patients with ICH treated with anticoagulation was obtained from a study by Fernando et al. We assumed that the mortality rate after an ICH was independent of type of anticoagulation treatment (warfarin vs NOAC). This assumption was based on a study by Skaistis et al showing no significant difference in fatal outcomes from bleedings, regardless of bleeding site and whether the patient was treated with NOAC or warfarin.

One-way sensitivity analysis: 0.59 - 2.36

Distribution in PSA: Lognormal 0.166, 0.0294)

Source: (10, 38)

### RR mortality post ICH

Base case: 2.0. We assumed that the long-term mortality rate after ICH was similar to the long-term mortality rate after IS. This assumption was based on a previous study by Sennfält et al showing that among 30-day survivors of IS and ICH, long-term survival was similar.

One-way sensitivity analysis: 1.0 - 4.0

Distribution in PSA: Lognormal (-0.693, 0.114)

Source: assumption and (39)

### Event rate myocardial infarction, warfarin treatment

Base case: 0.0112. The annual risk of myocardial infarction (MI) in patients with warfarin treatment was obtained from the ROCKET-AF study. The patients in ROCKET-AF had a CHADS₂ score of 3.46 and a mean age of 73, and the risk of MI in these patients was deemed reasonably comparable to the patients in the CDS-AF study. Similar event rates for MI in patients with AF and ongoing warfarin treatment have also been seen in some previous observational studies. (40)

One-way sensitivity analysis: 0.0056 - 0.0224

Distribution in PSA: Gamma (16, 0.0007)

Source: (11)

### RR of MI, untreated (compared to warfarin)

Base case: 1.96. The relative risk of MI in patients not treated with any anticoagulation compared to warfarin treated was obtained from a meta-analysis presented by Tawfik et al. The value presented in the base-case mixed-treatment comparison analysis was used.

One-way sensitivity analysis: 0.98 - 3.92

Distribution in PSA: Lognormal (0.673, 0.420)

Source: (9)

### RR of MI, NOAC treatment (compared to warfarin)

Base case: 0.97. The relative risk of MI in patients treated with NOAC compared to warfarin treated was obtained from a meta-analysis presented by Ruff et al.

One-way sensitivity analysis: 0.485 - 1.94

Distribution in PSA: Lognormal (-0.0305, 0.111)

Source: (5)

### Mortality rate first year, myocardial infarction

Base case: 0.206. The mortality rate in patients with MI was based on a study by Sulo et al. We calculated the mortality rate using data presented in the article 27,926/135,683 (Number of Deaths / Number of Patients at Risk).

One-way sensitivity analysis: 0.103- 0.412

Distribution in PSA: Gamma (16, 0.01286)

Source: (12)

### RR Mortality, post MI

Base case: 2,0. The long-term mortality rate after IS was obtained from a previous study by Smolina et al.

One-way sensitivity analysis: 1.0 - 4.0

Distribution in PSA: Lognormal (-0.693, 0.114)

Source: (13)

### Event rate systemic embolism, untreated

Base case: 0.00897. The annual risk of systemic embolism (SE) in patients not treated with anticoagulation was estimated using data from Friberg et al, and assuming that SE constituted 11.5 % of all embolic events, as previously described by Bekwelem et al. The Stroke or thromboembolism/100 years at risk in patients with CHA_2_DS_2_-VASc score 4 presented by Friberg et al was 0.078. The event rate for SE was approximated by the following calculation: 0.078 * 0.115 = 0.00897.

One-way sensitivity analysis: 0.00449 - 0.0179

Distribution in PSA: Gamma (16, 0.000560)

Source: (3, 14)

### RR of systemic embolism, warfarin treatment (compared to untreated)

Base case: 0.29. The relative risk of SE in patients treated with warfarin compared to patients not treated with any anticoagulation was estimated using data from a meta-analysis presented by Andersen et al.

One-way sensitivity analysis: 0.145 - 0.58

Distribution in PSA: Lognormal (-1.238, 0.666)

Source: (15)

### RR of systemic embolism, NOAC treatment (compared to warfarin)

Base case: 0.68. The relative risk of SE in patients treated with NOAC compared to patients treated with warfarin was obtained from a meta-analysis presented by Bruins Slot et al.

One-way sensitivity analysis: 0.34 - 1.36

Distribution in PSA: Lognormal (-0.386, 0.178)

Source: (16)

### Mortality rate first year, systemic embolism

Base case: 0.284. The mortality rate during the first year after SE was assumed to be similar to the mortality rate after IS, based on previously reported mortality data presented by Bekwelem et al that found no difference in short term morality (thirty-day mortality) between these groups.

One-way sensitivity analysis: 0.142 - 0.568

Distribution in PSA: Gamma (16, 0.0178)

Source: (6, 14)

### RR mortality post systemic embolism

Base case: 1.2754. The long-term mortality after SE was estimated by using data presented by Bekwelem et al. The authors found that the HR for mortality was 4.33 among patients with SE and 6.79 among patients with stroke compared with patients with neither of these events. We used this data to calculate the relative risk of long-term mortality after SE in our study. The following calculations were made: Long term mortality of SE compared to IS = 4.33/6.79 = 0.6377. Long term mortality after SE = 0.6377 * 2.0 (2.0 being the long-term mortality after IS used in our study) = 1.2754.

One-way sensitivity analysis: 0.638 - 2.551

Distribution in PSA: Lognormal (0.243, 0.176)

Source: (7, 14)

### Event rate major bleeding, warfarin

Base case: 0.0182. The annual risk of major bleeding (MB) in patients with warfarin treatment was estimated using data from Sjögren et al. In the paper, the authors present the event rates of gastrointestinal bleedings (0.7 %) and other bleedings (1.12 %) in patients with atrial fibrillation. We approximated the event rate of MBs in our study by summing these.

One-way sensitivity analysis: 0.0091 - 0.0364

Distribution in PSA: Gamma (16, 0.00114)

Source: (8)

### RR of major bleeding, untreated (compared to warfarin)

Base case: 0.60. The relative risk of MB in patients not treated with any anticoagulation compared to patients treated with warfarin was estimated using data from a meta-analysis presented by Hart RG et al. The authors present the relative risk reduction of major extracranial haemorrhage of warfarin vs control: -66 (-235 to 18). The risk of control vs warfarin was calculated as follows: = 1/1,66 = 0,6024.

One-way sensitivity analysis: 0.3 - 1.2

Distribution in PSA: Lognormal (-0.511, 0.362)

Source: (4)

### RR of major bleeding, NOAC treatment (compared to warfarin)

Base case: 0.977. The relative risk of MB in patients with NOAC treatment compared to patients treated with warfarin was estimated using data from Graham et al. The hazard ratios of major extracranial bleeding of Dabigatran, Rivaroxaban and Apixaban vs warfarin was found to be 1.04, 1.38 and 0.51, respectively. The relative risk of MB in patients with NOAC (pooled risk) compared to warfarin was approximated using the simple average of these: (1.04+1.38+0.51)/ 3 = 0.9767

One-way sensitivity analysis: 0.489 - 1.954

Distribution in PSA: Lognormal -0.0233, 0.0464)

Source: (17)

### Mortality rate major bleeding (untreated, NOAC, warfarin)

Base case: 0.03. The mortality rate after MB in patients treated with warfarin was estimated using data from Fang et al. Based on a study by Skaistis et al we assumed that the mortality rate of MB in patients with NOAC treatment was similar to the rate in patients treated with warfarin. There was a lack of studies directly informing on the mortality risk of MB in untreated patient compared to patients treated with anticoagulation. In the model we chose to assume that the mortality rate associated with MB in untreated was similar to patients treated with anticoagulation. In a previous study by Selak et al the authors presented case fatality rates of gastrointestinal bleedings in patients not treated with anticoagulation. These case fatality rates were somewhat similar to the 30-day mortality of the extracranial MBs in patients with warfarin treatment presented in the previously mentioned study by Fang et al (when comparing patients of similar age). Based on this we chose to assume that it was reasonable to use the same 1-year mortality rate.

One-way sensitivity analysis: 0.015 - 0.06

Distribution in PSA: Gamma (16, 0.00188)

Source: (18, 38, 41)

### Probability of not resuming anticoagulation after major bleeding

Base case: 0.53. After a MB event we estimated that 47 % of patients would resume anticoagulation therapy. We obtained data showing similar probabilities of resuming anticoagulation therapy from two different studies. To facilitate the modelling we assumed that all patients resuming anticoagulation therapy would restart NOAC therapy, even if the patient was on warfarin prior to the MB event. This assumption is somewhat in line with the clinical situation in our region, where NOAC is often used if the patient has a MB when on warfarin treatment.

One-way sensitivity analysis: 0.23 - 0.83. Only varied ± 0.3 instead of -50 % +200 % to ensure that the parameter remained between 0-1.

Distribution in PSA: Beta (6.99, 6.199)

Source: (19, 20)

## **Health related quality of life**

### Age specific quality of life

Base case: Age-specific. Age-specific QALY weights for the general population were sourced from a previously published study of the general population in Sweden presented by Burström et al.

One-way sensitivity analysis: Fixed

Distribution in PSA: Fixed

Source: (21)

### QALY weight stable atrial fibrillation

Base case: Same as general population. The same QALY weights were used for patients with AF in the model, since it has been shown that in the absence of comorbidities long-term AF has negligible impact on quality of life in elderly patient. In addition, as all patients in the model have AF, this assumption is not expected to have a large impact on the overall results of the analysis.

One-way sensitivity analysis: Fixed

Distribution in PSA: Fixed

Source: (22, 23)

### QALY-decrement anticoagulation treatment

Base case: 0.013. The QALY decrement for AF-patients treated with anticoagulation therapy was obtained from a previous study by Gage et al. Similar QALY weights were applied for warfarin and NOAC treated patients according to the findings of two previous studies presented by Monz et al and Van Miert et al.

One-way sensitivity analysis: 0.0065 - 0.026

Distribution in PSA: Gamma (16, 0.000813)

Source: (24, 42, 43)

### QALY-decrement ischemic stroke, first year

Base case: 0.160. EQ-5D data presented by Luengo-Fernandez et al was used to estimate the QALY-decrement the first year after IS. We compared EQ-5D utility in patients with IS with the reported EQ-5D utility of controls for stroke patients to calculate the decrement.

One-way sensitivity analysis: 0.08 - 0.32

Distribution in PSA: Gamma (16, 0.01)

Source: (25)

### QALY-decrement post ischemic stroke

Base case: 0.185. EQ-5D data presented by Luengo-Fernandez et al was used to estimate the long-term QALY-decrement post IS. We compared EQ-5D utility in patients with IS with the reported EQ-5D utility of controls for stroke patients to calculate the decrement.

One-way sensitivity analysis: 0.0925 - 0.37

Distribution in PSA: Gamma (16, 0.0116)

Source: (25)

### QALY-decrement ICH, first year

Base case: 0.2125. EQ-5D data presented by Luengo-Fernandez et al was used to estimate the QALY-decrement the first year after ICH. We compared EQ-5D utility in patients with ICH with the reported EQ-5D utility of controls for stroke patients to calculate the decrement.

One-way sensitivity analysis: 0.106 - 0.425

Distribution in PSA: Gamma (16, 0.0133)

Source: (25)

### QALY-decrement post ICH

Base case: 0.0688. EQ-5D data presented by Luengo-Fernandez et al was used to estimate the long-term QALY-decrement post ICH. We compared EQ-5D utility in patients with ICH with the reported EQ-5D utility of controls for stroke patients to calculate the decrement.

One-way sensitivity analysis: 0.034 - 0.138

Distribution in PSA: Gamma (16, 0.0043)

Source: (25)

### QALY-decrement all myocardial infarction states

Base case: 0.0627. A previously published cost-effectiveness analysis presented by Nikolic et al was used to obtain QALY-decrement for MI. The data presented in that study was based on EQ-5D data from the PLATO study. The authors of the study applied this decrement in a long-term model. We used the same decrement both for the first year after MI and for the long term in our model.

One-way sensitivity analysis: 0.0314 - 0.125

Distribution in PSA: Gamma (16, 0.00392)

Source: (26)

### QALY-decrement all systemic embolism states

Base case: 0.0508. A catalogue of EQ-5D Scores for the United Kingdom presented by Sullivan et al was used to estimate the QALY-decrement after SE. This data has previously been used in a cost-effectiveness analysis presented by Jacobs et al. We used the same decrement both for the first year after SE and for the long term in our model.

One-way sensitivity analysis: 0.0254 - 0.101

Distribution in PSA: Gamma (16, 0.00318)

Source: (27, 44)

### QALY-decrement major bleeding

Base case: 0.0174. A study presented by Wang et al was used to estimate the QALY decrement associated with major bleeding. We used the mean decrement of non-GI and GI bleeding (during the first year) to calculate the decrement used in our study.

One-way sensitivity analysis: 0.0087 - 0.0348

Distribution in PSA: Gamma (16, 0.00109)

Source: (28)

## Costs used in Disease progression model

### Stroke, first year (ischemic + ICH)

Base case: €19,198. The cost of stroke was obtained from a report of stroke costs in Sweden published by Ghatnekar and Steen Karlsson. We only considered direct costs since very few of the patients were assumed to be working due to high age. We used the average costs of men and women in the analysis. We assumed similar costs for IS and ICH in our model.

One-way sensitivity analysis: €9599- 38,396

Distribution in PSA: Gamma (16, 1199.875)

Source: (29)

### Post stroke (ischemic + ICH)

Base case: €3796. Cost of stroke was obtained from a report of stroke costs in Sweden published by Ghatnekar and Steen Karlsson. We only considered direct costs since very few of the patients were assumed to be working due to high age. We used the average costs of men and women in the analysis. To calculate the long-term costs, post stroke we used the average costs of year 2-4 after stroke. We assumed similar costs for IS and ICH in our model.

One-way sensitivity analysis: €1898 - 7592

Distribution in PSA: Gamma (16, 237.25)

Source: (29)

### Myocardial infarction, first year

Base case: €14,850. Excess costs of MI during the first year after event were estimated using data from a nationwide Swedish study of patients with MI published by Janzon et al. We included costs of hospitalizations, outpatient care visits, and the use of pharmaceuticals, but excluded primary care visits since those costs did not increase clearly after the MI event.

One-way sensitivity analysis: €7425 – 29,700

Distribution in PSA: Gamma (16, 928.125)

Source: (30)

### Post myocardial infarction

Base case: €1867. Data from Janzon et al were used to estimate the long-term costs after MI. We included costs of hospitalizations, outpatient care visits, and the use of pharmaceuticals, but excluded primary care visits since those costs did not increase clearly after the MI event. To calculate the long-term cost, we used the average costs of year 2-6 after MI.

One-way sensitivity analysis: €934 - 3734

Distribution in PSA: Gamma (16, 116.6875)

Source: (30)

### Systemic embolism, first year

Base case: €4664. The first-year cost after SE was obtained from a cost-effectiveness analysis published by Lanitis et al.

One-way sensitivity analysis: €2332 - 9328

Distribution in PSA: Gamma (16, 291.5)

Source: (31)

### Post systemic embolism

Base case: €864. There was a lack of studies, from relevant clinical settings, informing on the long-term cost of SE. We therefore assumed, conservatively, that the long-term cost of SE was similar to the maintenance cost of NOAC treatment.

One-way sensitivity analysis: €432 - 1728

Distribution in PSA: Gamma (16, 54)

Source: Assumption

### Major bleeding

Base case: €2713. The cost of MB was obtained from a cost-effectiveness analysis published by Lanitis et al. We calculated the average cost of a MB by assuming that the proportion of GI bleedings vs other MBs was the same as the proportion found in a previous study presented by Sjögren et al.

One-way sensitivity analysis: €1356.5 - 5426

Distribution in PSA: Gamma (16, 169.562)

Source: (8, 31)

### Warfarin (yearly cost)

Base case: €669. The annual cost of warfarin treatment was estimated using the lowest retail drug prices available in Sweden in 2023. In addition, we also added an estimate of the annual costs of outpatient follow-ups including annual lab tests associated with the warfarin treatment. These costs were estimated by using data from a previous report published by the Swedish council on Health Technology Assessment (CMT) and by sourcing data from price lists published by the Sydöstra sjukvårdsregionen and Region Östergötland.

One-way sensitivity analysis: €334.5 - 1338

Distribution in PSA: Gamma (16, 41.8125)

Source: (32-35)

### NOAC (yearly cost)

Base case: €864. The annual cost of NOAC treatment was estimated using the lowest retail drug prices available in Sweden in 2023. We used the average cost of Apixaban and Rivaroxaban, since those two NOACs were the most frequently used in our region. In addition, we also added an estimate of the annual costs of outpatient follow-ups including annual lab tests associated with NOAC treatment. We assumed that NOAC treatment was associated with one annual outpatient visit and one annual blood sampling. The costs of outpatient visits and lab tests were obtained from price lists published by the Sydöstra sjukvårdsregionen and Region Östergötland.

One-way sensitivity analysis: €432 - 1728

Distribution in PSA: Gamma (16, 54)

Source: (33-35)

### CDS (total one-time cost per patient)

Base case: €2.996. Data obtained from the CDS-AF study was used to determine the cost of using the CDS. We estimated the average time needed for a primary care physician to manage a CDS pop-up to 15 minutes, by using our best clinical judgment. We estimated the time required to initiate anticoagulation therapy and informing the patient to 30 minutes. The validity of these estimates was verified with several external primary care physicians, not part of the study group. The unit costs applied to the working hours consumed was estimated using the average Swedish primary care physician salary in 2023 obtained from the Swedish Confederation of Professional Associations (SACO), including employer's contributions. We also registered the annual fees associated with using the CDS. This information was obtained directly from the information technology department of Region Östergötland. The one-time cost for development and implementation of the CDS was not incorporated into the disease progression model in the base case. We estimated these costs to approximately €185 000. These costs included working hours consumed by coding, testing, and implementing of the CDS, but they did not take into account working hours of the individuals in the research group.

One-way sensitivity analysis: €1.50- 5.99

Distribution in PSA: The one-time cost of per patient for using the CDS was a sum of several different parameters that were all varied in the PSA

Source: Estimate + (45)

# References

1. Karlsson LO, Nilsson S, Bang M, Nilsson L, Charitakis E, Janzon M. A clinical decision support tool for improving adherence to guidelines on anticoagulant therapy in patients with atrial fibrillation at risk of stroke: A cluster-randomized trial in a Swedish primary care setting (the CDS-AF study). PLoS Med. 2018;15(3):e1002528.

2. Socialstyrelsen. Statistikdatabas för läkemedel 2024. Available from: <https://www.socialstyrelsen.se/statistik-och-data/statistik/statistikdatabasen/>

3. Friberg L, Rosenqvist M, Lip GY. Evaluation of risk stratification schemes for ischaemic stroke and bleeding in 182 678 patients with atrial fibrillation: the Swedish Atrial Fibrillation cohort study. Eur Heart J. 2012;33(12):1500-10.

4. Hart RG, Pearce LA, Aguilar MI. Meta-analysis: antithrombotic therapy to prevent stroke in patients who have nonvalvular atrial fibrillation. Ann Intern Med. 2007;146(12):857-67.

5. Ruff CT, Giugliano RP, Braunwald E, Hoffman EB, Deenadayalu N, Ezekowitz MD, et al. Comparison of the efficacy and safety of new oral anticoagulants with warfarin in patients with atrial fibrillation: a meta-analysis of randomised trials. The Lancet. 2014;383(9921):955-62.

6. Vinding NE, Kristensen SL, Rørth R, Butt JH, Østergaard L, Olesen JB, et al. Ischemic Stroke Severity and Mortality in Patients With and Without Atrial Fibrillation. J Am Heart Assoc. 2022;11(4):e022638.

7. Fang MC, Go AS, Chang Y, Borowsky LH, Pomernacki NK, Udaltsova N, et al. Long-term survival after ischemic stroke in patients with atrial fibrillation. Neurology. 2014;82(12):1033-7.

8. Sjogren V, Grzymala-Lubanski B, Renlund H, Friberg L, Lip GY, Svensson PJ, et al. Safety and efficacy of well managed warfarin. A report from the Swedish quality register Auricula. Thromb Haemost. 2015;113(6):1370-7.

9. Tawfik A, Bielecki J, Krahn M, Dorian P, Hoch J, Boon H, et al. Systematic review and network meta-analysis of stroke-prevention treatments in patients with atrial fibrillation. Clin Pharmacol. 2016;8:93-107.

10. Fernando SM, Qureshi D, Talarico R, Tanuseputro P, Dowlatshahi D, Sood MM, et al. Intracerebral Hemorrhage Incidence, Mortality, and Association With Oral Anticoagulation Use. Stroke. 2021;52(5):1673-81.

11. Patel MR, Mahaffey KW, Garg J, Pan G, Singer DE, Hacke W, et al. Rivaroxaban versus Warfarin in Nonvalvular Atrial Fibrillation. N Engl J Med. 2011;365(10):883-91.

12. Sulo G, Igland J, Sulo E, Øverland S, Egeland GM, Vollset SE, et al. Mortality following first-time hospitalization with acute myocardial infarction in Norway, 2001–2014: Time trends, underlying causes and place of death. Int J Cardiol. 2019;294:6-12.

13. Smolina K, Wright FL, Rayner M, Goldacre MJ. Long-Term Survival and Recurrence After Acute Myocardial Infarction in England, 2004 to 2010. Circ Cardiovasc Qual Outcomes. 2012;5(4):532-40.

14. Bekwelem W, Connolly SJ, Halperin JL, Adabag S, Duval S, Chrolavicius S, et al. Extracranial Systemic Embolic Events in Patients With Nonvalvular Atrial Fibrillation. Circulation. 2015;132(9):796-803.

15. Andersen LV, Vestergaard P, Deichgraeber P, Lindholt JS, Mortensen LS, Frost L. Warfarin for the prevention of systemic embolism in patients with non-valvular atrial fibrillation: a meta-analysis. Heart. 2008;94(12):1607-13.

16. Bruins Slot KM, Berge E. Factor Xa inhibitors versus vitamin K antagonists for preventing cerebral or systemic embolism in patients with atrial fibrillation. Cochrane Database Syst Rev. 2018;3(3):CD008980.

17. Graham DJ, Baro E, Zhang R, Liao J, Wernecke M, Reichman ME, et al. Comparative Stroke, Bleeding, and Mortality Risks in Older Medicare Patients Treated with Oral Anticoagulants for Nonvalvular Atrial Fibrillation. Am J Med. 2019;132(5):596-604 e11.

18. Fang MC, Go AS, Chang Y, Hylek EM, Henault LE, Jensvold NG, et al. Death and Disability from Warfarin-Associated Intracranial and Extracranial Hemorrhages. Am J Med. 2007;120(8):700-5.

19. Qureshi W, Mittal C, Patsias I, Garikapati K, Kuchipudi A, Cheema G, et al. Restarting anticoagulation and outcomes after major gastrointestinal bleeding in atrial fibrillation. Am J Cardiol. 2014;113(4):662-8.

20. Hernandez I, Zhang Y, Brooks MM, Chin PK, Saba S. Anticoagulation Use and Clinical Outcomes After Major Bleeding on Dabigatran or Warfarin in Atrial Fibrillation. Stroke. 2017;48(1):159-66.

21. Burström K, Johannesson M, Diderichsen F. A comparison of individual and social time trade-off values for health states in the general population. Health Policy. 2006;76(3):359-70.

22. Reynolds MR, Lavelle T, Essebag V, Cohen DJ, Zimetbaum P. Influence of age, sex, and atrial fibrillation recurrence on quality of life outcomes in a population of patients with new-onset atrial fibrillation: The Fibrillation Registry Assessing Costs, Therapies, Adverse events and Lifestyle (FRACTAL) study. Am Heart J. 2006;152(6):1097-103.

23. Roalfe AK, Bryant TL, Davies MH, Hackett TG, Saba S, Fletcher K, et al. A cross-sectional study of quality of life in an elderly population (75 years and over) with atrial fibrillation: secondary analysis of data from the Birmingham Atrial Fibrillation Treatment of the Aged study. Europace. 2012;14(10):1420-7.

24. Gage BF, Cardinalli AB, Owens DK. The Effect of Stroke and Stroke Prophylaxis With Aspirin or Warfarin on Quality of Life. Arch Intern Med. 1996;156(16):1829-36.

25. Luengo-Fernandez R, Gray AM, Bull L, Welch S, Cuthbertson F, Rothwell PM. Quality of life after TIA and stroke: Ten-year results of the Oxford Vascular Study. Neurology. 2013;81(18):1588-95.

26. Nikolic E, Janzon M, Hauch O, Wallentin L, Henriksson M. Cost-effectiveness of treating acute coronary syndrome patients with ticagrelor for 12 months: results from the PLATO study. Eur Heart J. 2013;34(3):220-8.

27. Sullivan PW, Slejko JF, Sculpher MJ, Ghushchyan V. Catalogue of EQ-5D Scores for the United Kingdom. Med Decis Making. 2011;31(6):800-4.

28. Wang K, Li H, Kwong WJ, Antman EM, Ruff CT, Giugliano RP, et al. Impact of Spontaneous Extracranial Bleeding Events on Health State Utility in Patients with Atrial Fibrillation: Results from the ENGAGE AF-TIMI 48 Trial. J Am Heart Assoc. 2017;6(8):e006703.

29. Ghatnekar O, Steen Karlsson K. Kostnader för insjuknande i stroke år 2009 - En incidensbaserad studie. IHE Rapport. Institutet för Hälso- och Sjukvårdsekonomi; 2012.

30. Janzon M, Henriksson M, Hasvold P, Hjelm H, Thuresson M, Jernberg T. Long-term resource use patterns and healthcare costs after myocardial infarction in a clinical practice setting: results from a contemporary nationwide registry study. Eur Heart J Qual Care Clin Outcomes. 2016;2(4):291-8.

31. Lanitis T, Kongnakorn T, Jacobson L, De Geer A. Cost-effectiveness of Apixaban versus Warfarin and Aspirin in Sweden for Stroke Prevention in Patients with Atrial Fibrillation. Thromb Res. 2014;134(2):278-87.

32. Tandvårds- och läkemedelsförmånsverket (TLV). Priser och beslut 2024. Available from: <https://www.tlv.se/beslut/sok-priser-och-beslut-i-databasen.html>.

33. Davidsson T, Husberg M, Janzon M, Levin L-Å. Dabigatran för att förebygga stroke vid förmaksflimmer. Swedish council on Health Technology Assessment; 2011.

34. Sydöstra sjukvårdsregionen. Priser och ersättningar för Sydöstra sjukvårdsregionen 2023 - justering. 2023.

35. Region Östergötland. Prislista Klinisk kemi 2024. Available from: <https://vardgivare.regionostergotland.se/vgw/kunskapsstod/medicinsk-diagnostik/laboratoriemedicin/prislistor-laboratoriemedicin>.

36. Tandvårds- och Läkemedelsförmånsverket (TLV). Läkemedelsförmånsnämndens allmänna råd om ekonomiska utvärderingar LFNAR 2003:2. 2003.

37. Statistics Sweden. Statistical database, Life table by sex and age 2024. Available from: <https://www.statistikdatabasen.scb.se/pxweb/en/ssd/START__BE__BE0101__BE0101I/LivslangdEttariga/>.

38. Skaistis J, Tagami T. Risk of Fatal Bleeding in Episodes of Major Bleeding with New Oral Anticoagulants and Vitamin K Antagonists: A Systematic Review and Meta-Analysis. PLoS One. 2015;10(9):e0137444.

39. Sennfält S, Norrving B, Petersson J, Ullberg T. Long-Term Survival and Function After Stroke. Stroke. 2019;50(1):53-61.

40. Violi F, Soliman EZ, Pignatelli P, Pastori D. Atrial Fibrillation and Myocardial Infarction: A Systematic Review and Appraisal of Pathophysiologic Mechanisms. J Am Heart Assoc. 2016;5(5):e003347.

41. Selak V, Kerr A, Poppe K, Wu B, Harwood M, Grey C, et al. Annual Risk of Major Bleeding Among Persons Without Cardiovascular Disease Not Receiving Antiplatelet Therapy. JAMA. 2018;319(24):2507-20.

42. Monz BU, Connolly SJ, Korhonen M, Noack H, Pooley J. Assessing the impact of dabigatran and warfarin on health-related quality of life: results from an RE-LY sub-study. Int J Cardiol. 2013;168(3):2540-7.

43. Van Miert JHA, Kooistra HAM, Veeger NJGM, Westerterp A, Piersma-Wichers M, Meijer K. Quality of life after switching from well-controlled vitamin K antagonist to direct oral anticoagulant: Little to GAInN. Thromb Res. 2020;190:69-75.

44. Jacobs MS, Kaasenbrood F, Postma MJ, van Hulst M, Tieleman RG. Cost-effectiveness of screening for atrial fibrillation in primary care with a handheld, single-lead electrocardiogram device in the Netherlands. Europace. 2018;20(1):12-8.

45. Swedish Confederation of Professional Association. Salery statistics 2023. Available from: <https://lonesok.saco.se/>.
